# Multiple sequence alignment based on deep reinforcement learning with self-attention and positional encoding

**DOI:** 10.1093/bioinformatics/btad636

**Published:** 2023-10-19

**Authors:** Yuhang Liu, Hao Yuan, Qiang Zhang, Zixuan Wang, Shuwen Xiong, Naifeng Wen, Yongqing Zhang

**Affiliations:** School of Computer Science, Chengdu University of Information Technology, Chengdu 610225, China; School of Computer Science, Chengdu University of Information Technology, Chengdu 610225, China; School of Computer Science, Chengdu University of Information Technology, Chengdu 610225, China; College of Electronics and Information Engineering, Sichuan University, Chengdu 610065, China; School of Computer Science, Chengdu University of Information Technology, Chengdu 610225, China; School of Mechanical and Electrical Engineering, Dalian Minzu University, Dalian 116600, China; School of Computer Science, Chengdu University of Information Technology, Chengdu 610225, China

## Abstract

**Motivation:**

Multiple sequence alignment (MSA) is one of the hotspots of current research and is commonly used in sequence analysis scenarios. However, there is no lasting solution for MSA because it is a Nondeterministic Polynomially complete problem, and the existing methods still have room to improve the accuracy.

**Results:**

We propose Deep reinforcement learning with Positional encoding and self-Attention for MSA, based on deep reinforcement learning, to enhance the accuracy of the alignment Specifically, inspired by the translation technique in natural language processing, we introduce self-attention and positional encoding to improve accuracy and reliability. Firstly, positional encoding encodes the position of the sequence to prevent the loss of nucleotide position information. Secondly, the self-attention model is used to extract the key features of the sequence. Then input the features into a multi-layer perceptron, which can calculate the insertion position of the gap according to the features. In addition, a novel reinforcement learning environment is designed to convert the classic progressive alignment into progressive column alignment, gradually generating each column’s sub-alignment. Finally, merge the sub-alignment into the complete alignment. Extensive experiments based on several datasets validate our method’s effectiveness for MSA, outperforming some state-of-the-art methods in terms of the Sum-of-pairs and Column scores.

**Availability and implementation:**

The process is implemented in Python and available as open-source software from https://github.com/ZhangLab312/DPAMSA.

## 1 Introduction

Multiple sequence alignment (MSA) aims to align similar or homologous regions between multiple sequences at the least cost of minimum insertion of gaps. MSA has been widely used in biological sequence analysis (Wang *et al.* 2020), structure prediction, phylogenetic analysis, and other analysis scenarios (Kandeel *et al.* 2022). However, MSA is a Nondeterministic Polynomially complete problem; its temporal and spatial complexity increases exponentially with the rise of sequences (Chatzou *et al.* 2016). Nevertheless, an effective MSA method can certainly facilitate researchers to deal with many sequences and reduces the cost (Zhang *et al.* 2022).

Progressive alignment is one of the most commonly applied to MSA, such as ClustalW (Thompson *et al.* 1994), T-Coffee (Notredame *et al.* 2000), and Kalign (Lassmann 2019). In addition, Bhat *et al.* (2017) developed TM-Aligner, a transmembrane protein sequence alignment program based on progressive alignment. Maiolo *et al.* (2021) proposed ProPIP in 2021, a method based on Poisson Indel Process model and progressive alignment. Garriga *et al.* (2021) proposed a method combining progressive alignment and regression algorithm starting from the farthest sequence. Their approach can significantly improve the alignment accuracy in large-scale sequences, meanwhile. Therefore some researchers combine iterative algorithms with improving accuracy based on progressive alignments, such as MAFFT (Katoh *et al.* 2002), MUSCLE (Edgar 2004), and PASTA (Mirarab *et al.* 2015). Libin *et al.* (2019) presented a technique based on iterative reference-guided–VIRULIGN in 2019. Moshiri *et al.* proposed a method similar to VIRULIGN, ViralMSA (Moshiri 2021). ViralMSA is many orders of magnitude faster than VIRULIGN in speed, and the memory consumption is also lower. In addition, some efforts have been made to apply the heuristics algorithm in 2019. Hussein *et al.* (2019) used the flower pollination algorithm to solve the MSA. In 2020, Chowdhury and Garai proposed an alignment method based on a genetic algorithm–BSAGA (Chowdhury and Garai 2020).

In addition to the above classic algorithms, some researchers tried to introduce reinforcement learning to solve the problem of MSA. For example, Mircea *et al.* (2015) proposed an MSA method based on Q-Learning in 2015. The most prominent feature of this method is to replace the guide tree with Q-learning and use Q-learning to guide the construction of alignment. Then Reza *et al.* improved Mircea’s work and proposed an A3C (Asynchronous Advantage Actor Critic)-based method (Jafari *et al.* 2019). In addition, Ramakrishnan *et al.* (2018) presented RLALIGN based on the A3C model in 2018. However, the above methods are only improved based on progressive alignment and do not use the characteristics of the sequence itself. Besides, the model is also difficult to converge.

To address the above problems, we first proposed Deep reinforcement learning with Positional encoding and self-Attention for MSA (DPAMSA), an MSA method combining natural language processing technology and deep reinforcement learning (DRL) in MSA (Takase and Okazaki 2019, Zhang *et al.* 2020, Galassi *et al.* 2021) Moreover, we also designed a new reinforcement learning environment to improve accuracy and reliability. The former is mainly based on progressive column alignment, and the sub-alignment of each column is calculated step by step. Then all sub-alignments are spliced into a complete alignment. The method particularly inserts a gap according to the current sequence state Deep Q Network (DQN) is the DRL model The model’s Q network is divided into positional encoding, self-attention, and multi-layer perceptron. Finally, comparative experiments show that DPAMSA can achieve higher or similar accuracy on most datasets compared with MAFFT (Katoh *et al.* 2002), ClustalW (Thompson *et al.* 1994), MSAProbs (Liu *et al.* 2010), T-Coffee (Notredame *et al.* 2000), MUSCLE (Edgar 2004), PronCons (Libin *et al.* 2019), and Clustal Ω (Sievers *et al.* 2011).

## 2 Materials and methods

### 2.1 Preliminaries

Given a dataset *S* contains *s* sequences, the corresponding alignment is *CA*. However, there is no entirely correct biological evolution model, and we cannot judge whether the alignment *CA* conforms to the law of natural evolution. Therefore, the alignment *CA* of dataset *S* may not be unique for the current research. There may be multiple or even countless results, recorded as the set ${\left\{CA_{i}\right\}}_{i=1}^{\infty }$ In MSA, and there are corresponding evaluation criteria for any $CA_{i}$. The SP (Sum-of-Pairs) score is the most commonly used, calculated as follows (Altschul and Lipman 1989).


(1) $$SP(CA)=\sum_{k=1}^{\mathrm{len}(CA)}\sum_{i=1}^{n-1}\sum_{j=i+1}^{n}p(CA^{i,k},CA^{j,k})$$

where len(CA) represents the length of a single sequence in the alignment *CA*, and $CA^{i,k}$ represents the *k*th deoxynucleotide of the *i*th sequence. The function p(x,y) is used to calculate the matching score of deoxynucleotides *x* and *y*, and the calculation equation is shown in Equation 2.


(2) $$p(x,y)=\{\begin{array}{cc} -4 & x=\mathrm{gap}|y=\mathrm{gap}\\ 4 & x=y\\ -4 & x\neq y \end{array}$$

The calculation basis of this function mainly includes the following three kinds: (i) Gap penalty: when either *x* or *y* is a gap, the score is −4. (ii) Matching score: when *x* and *y* are the same, the score is 4. (iii) Mismatch penalty: when *x* and *y* are different, the score is −4. It should be noted that the values of the three scores are not fixed, but the reward score must be greater than the penalty score. During the calculation, the first judge is a gap in *x* and *y*, and then judge the matching of *x* and *y*. Therefore, the SP score is the sum of the scores of all paired deoxynucleotides in the alignment.

This study aims to find the alignment with the highest SP score. Therefore, the overall objective function can be expressed as.


(3) $$\max(SP(CA)),CA\in {\left\{CA_{i}\right\}}_{i=1}^{\infty }$$

Another commonly used evaluation indicator is column score (CS). For the *i*th column in the aligned area described above, the score $C_{i}=1$ if all residues are the same; otherwise, $C_{i}=0$. The CS Score is:


(4) $$CS=\sum_{i=1}^{M}C_{i}/M$$

### 2.2 Model description

**Figure 1.**
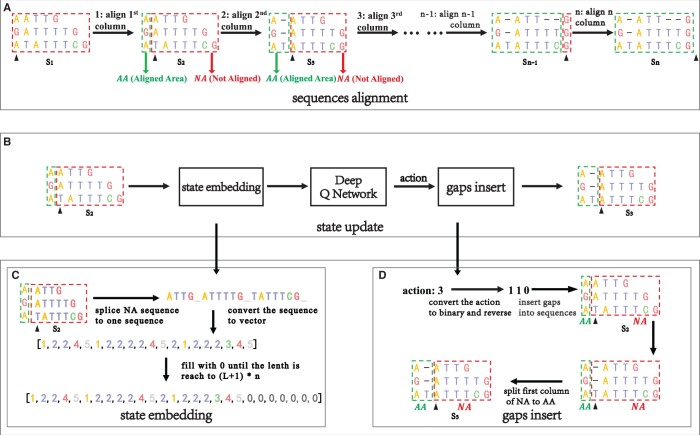
This is a case of DPAMSA. (A) The process of DPAMSA sequence alignment, column-by-column alignment, and each column alignment results in a change in sequence state. (B) The specific process of sequence state update. (C) The process of state embedding. (D) The process of gaps insert.

Figure 1 shows the detailed process of Sequence alignment. Figure 1A shows the process of sequence alignment on a column-by-column basis, and the status of the sequence is updated once each column alignment is completed. Figure 1B shows in detail the update process of the state from $S_{2}$ to $S_{3}$. Figure 1C and D respectively shows a detailed introduction about state embedding and gaps insert in Figure 1B.

**Sequence alignment**: Figure 1A shows the process of sequence alignment. Given a sequence *S*, sequence alignment is performed column by column. The sequence state changes when a column is aligned, such as $S_{1}$, $S_{2}$, and $S_{3}$, until the last column alignment is completed. During the alignment process, the columns that have completed sequence alignment are framed with green boxes, denoted as *AA* (Aligned Area). In contrast, the columns that have not completed the alignment are surrounded by a red box, denoted as *NA* (Not Aligned Area). The black arrow points to the current alignment column.

**State update**: Figure 1B shows the process of sequence state update from $S_{2}$ to $S_{3}$. First, the not aligned sequence in $S_{2}$ is embedded into an embedding sequence vector, passed the vector into the deep Q network, gets an action value through the deep Q network, then inserts gaps into the current aligned column according to the action value.

**State embedding**: Figure 1C shows the process of state embedding. All sequences in *NA* are spliced into a long sequence and then converted into the corresponding sequence integer vector. Taking the state $S_{2}$ in Fig. 1A as an example, a dataset contain three deoxynucleotide sequence, and its current unaligned part *NA* is [*ATTG*, *ATTTTG*, *TATTTCG*]. According to the above definition, each sequence in the *NA* is spliced into a long sequence, which is expressed as “ATTG_ATTTTG_TATTTCG_.” Then, the long sequence is converted into state vector [1,2,2,4,5,1,2,2,2,2,4,5,2, 1,2,2,2,3,4,5]. After each column alignment, the length of the state vector will be reduced, which is not suitable for the input of the neural network. Therefore, this method fixes the length of the state vector as n×(L+1), empty part in the vector is filled with the number 0 *L* is the maximum length of the three aligned sequences, and *n* is the number of aligned sequences. Therefore, the final representation of the initial state is [1, 2, 2, 4, 5, 1, 2, 2, 2, 2, 4, 5, 2, 1, 2, 2, 2, 3, 4, 5, 0, 0, 0, 0, 0, 0, 0].

**Gaps insertion**: Figure 1D shows the process of sequence gaps insert. First, the action value 3 is binary encoded as 011 and reversed to 110. Obtain the corresponding values from high to low (1, 1, 0). No change occurs when the value is 1, insert gap, and the value is 0. So the column pointed by the arrow in $S_{2}$, the first and second columns are inserted gaps, while the third column is unchanged, resulting in state $S_{3}$.

### 2.3 Environment in reinforcement learning

The environment in reinforcement learning mainly includes the agent, state, action, and reward functions. Their definitions are as follows (Kaelbling *et al.* 1996).

**Agent**: Generally, the agent is regarded as an entity with a specific decision-making ability. In reinforcement learning, agents can continuously interact with the environment to adjust or optimize their strategies. The behavior characteristics of the DRL model are the same as that of the agent. Therefore, the DRL model is an agent in this study.

**State**: Represented by *s*, the state in this study is the state integer vector, such as the state $S_{2}\;[1,2,2,4,5,1,2,2,2,2,4,5,2,1,2,2,2,\;3,4,5,0,0,0,0,0,0,0]$ in Fig. 1C.

**Action**: Represented by *a*. Action is an integer value used to control the insertion of gaps at the corresponding position of the current alignment column in the sequence Fig. 1D, the sequence alignment state changed from $S_{2}$ to $S_{3}$ by gaps insert. The action value is 3, using binary encoding and reversed to become 110, according to the rule of 1 insertion gaps and 0 no operation, the insertion gaps operation is performed on the first and second columns of the current alignment column indicated by the arrow, and the third column is not changed.

**Episode**: The sequence generated by the interaction between the agent and the environment is called an episode, which records all the state transition processes from the initial state to the end state and is shown below:


(5) $$\mathrm{episode}=s_{0}\;\overset{a_{0}}{\to }\;s_{1}\;\overset{a_{1}}{\to }\;\cdots s_{t}\cdots \overset{a_{T-2}}{\to }\;s_{T-1}\;\overset{a_{T-1}}{\to }\;s_{T}$$

where $s_{t}$ is the state of the environment at time-step *t*, $a_{t}$ is the environment in state $s_{t}$ the action to be performed. *T* is the termination time-step, meaning that after time-step *T*, the agent and the environment no longer interact, which marks the end of the current episode. There are two cases for termination time in this study: normal termination and abnormal termination. Normal termination occurs when all sequences are aligned, the length of all sequences in $NA_{T}$ is 0. The mathematical expression corresponding to the termination condition is $\sum_{i=1}^{n}len(NA_{T}^{i})=0$, where $NA_{T}^{i}$ means the *i*th sequence in $NA_{T}$. when there is a sequence with length 0 in $NA_{T}$, and its corresponding action vector is 0, it indicates abnormal termination. Due to there being an empty sequence in $NA_{T-1}$, it means that it is no longer possible to separate the complete column from $NA_{T-1}$. At this time, the current episode should be ended to avoid unpredictable errors. The corresponding mathematical form of abnormal termination condition is $\exists \mathrm{seq}\in NA_{T-1}$, len(seq) = 0.

**Reward**: Represented by *r*. The reward value used in the method is directly represented by the SP score in the first column of *NA*. The formula is as follows:


(6) $$r_{t}=\{\begin{array}{cc} -r_{\max} & \exists \mathrm{seq}\in NA_{T-1},\\ & \mathrm{len}(\mathrm{seq})=0\\ \sum_{i=1}^{n-1}\sum_{j=i+1}^{n}p(NA_{t}^{i,1},NA_{t}^{j,1}) & \mathrm{otherwise} \end{array}$$

where $r_{\max}$ is the largest *SP* score in a single column, all nucleotides in this column are the same. Its value is $\frac{4n\times (n-1))}{2}$. When the termination time step is reached, the current episode should be ended immediately. The returned reward value $-r_{\max}$ Returning a negative reward value can reduce the cumulative reward value with the abnormal termination state as the final state to reduce the weight of the bad action. In other cases, including the normal termination state, the reward value is $r_{t}$ is determined by the formula $\sum_{i=1}^{n-1}\sum_{j=i+1}^{n}p(NA_{t}^{i,1},NA_{t}^{j,1})$, where $NA_{t}^{i,1}$ represents the first nucleotide of *i*th sequence in $NA_{t}$.

### 2.4 Method

**Figure 2.**
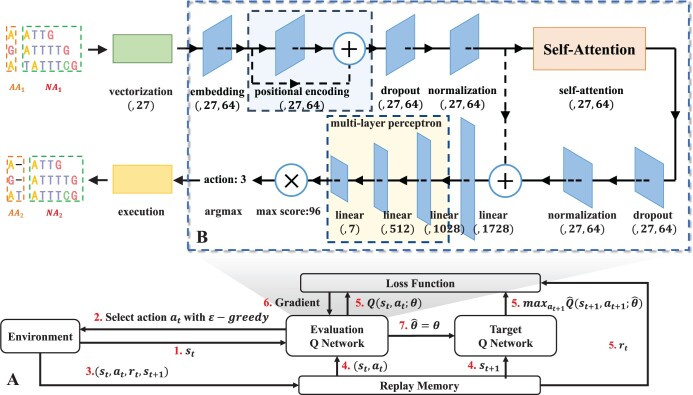
(A) The main model used in DPAMSA. (B) The overall structure of the Q Network. The network is mainly divided into three parts. The first part is positional encoding, the second is the self-attention model, and the third is a multi-layer perceptron. After the multi-layer perceptron outputs, the output vector needs to be multiplied by a theoretical maximum of Q value (96 in the figure) to obtain the Q value vector. Finally, take the index of the maximum Q value as the action. The structure of the self-attention model.

The whole DRL design is shown in our proposed method in Fig. 2A. First, transfer the original sequence *S* to the environment. The environment will obtain the initial state $S_{1}$ based on the sequence *S*. Then, according to the ϵ-greedy policy, receive the corresponding action value and return it to the environment; this is repeated until the Sequence alignment is completed. The ϵ-greedy strategy randomly gets action values with probability 1−ϵ and obtains action values through the validation Q network with probability Fig. 2B shows the composition of deep Q network (DQN) (Mnih *et al.* 2015), which includes three-part: *Self-Attention mechanism*, *Positional encoding* and *Multi-Layer perceptron*.

- **Self-Attention mechanism**: The main function of this part is to extract key features from the current state of the sequences, which is very important to enhance the accuracy of the alignment and the reliability of the model.
- **Positional encoding**: Since the self-attention mechanism is to lose the position information of the deoxynucleotides in the sequence, the purpose of positional encoding is to retain the position information before extracting features.
- **Multi-Layer perceptron**: This part is located after the self-attention mechanism. It mainly calculates the Q values of all actions at the current state through a multi-layer full connection layer based on the extracted features.

### 2.5 Self-attention

The attention mechanism can map the query, key, and value vector into the output vector to extract features. To improve the accuracy and the model’s reliability, we introduce the attention mechanism into the method to make the model “pay attention” to the correlation of each deoxynucleotide in the input sequence. The primary step of the attention mechanism is to calculate the similarity between *Q* and *K*, then multiply the similarity and *V* to achieve the weight allocation of the input vector. Generally, the expression of the attention mechanism is as follows.


(7) $$\begin{array}{c} \begin{array}{cc} \mathrm{attention}(Q,K,V) & =\frac{\;\text{exp}(s(Q,K))}{\sum (s(Q,K))}\cdot V \end{array}\\ \begin{array}{c} =\mathrm{softmax}(s(Q,K)) \end{array} \end{array}$$

where, *Q*, *K*, and *V* correspond to *query*, *key*, and *value* vectors, respectively. s(Q,K) is an important part of the attention mechanism. It determines the types of attention mechanisms, such as additive attention, dot product attention, and scaled dot product attention (Niu *et al.* 2021). In DPAMSA, we use the scaled dot product attention (as shown in Supplementary Fig. S1), and its corresponding s(Q,K) expression is as follows.


(8) $$s(Q,K)=\frac{Q\cdot K^{T}}{\sqrt{d_{k}}}$$

where $d_{k}$ is the vector dimension of *Q* and *K*. In the similarity calculation of *Q* and *K* by the other two attention mechanisms, additive attention uses the forward feedback network with a single hidden layer. The dot product attention directly multiplies the two vectors of *Q* and *K*. Since dot product attention can be implemented by highly optimized matrix multiplication. Scale dot product attention is calculated similarly to dot product attention, except that it divides the product of two vectors by $\sqrt{d_{k}}$ (Vaswani *et al.* 2017), where $d_{k}$ is the dimension of vector *Q* and *K*. The purpose is to prevent the variance of vector $Q\cdot K^{T}$ from being too large, resulting in the model’s attention being focused on a particular area. At the same time, scaling the product results can appropriately improve the model’s generalization ability.

In addition to the attention(Q,K,V) calculation, the source of *Q*, *K*, and *V* vectors is significant. These three vectors come from the same input vector in DPAMSA. The scale dot product attention is also called self-attention. The attention structure is shown in Fig. 2C. The input is the embedded vector of the sequence vector. In the first step, the embedded vector is input into three linear neural networks to obtain *Q*, *K*, and *V* vectors. Then, calculate $\mathrm{softmax}(\frac{Q\cdot K^{T}}{\sqrt{d_{k}}})$ and multiply the result with vector *V*. Finally, the product is input into the linear neural network to restore the size of the vector to the size of the input (Zhang *et al.* 2019).

### 2.6 Positional encoding

Because there is no convolution and recurrent mechanism in the model, the model can not capture the structure information of sequence and the position information of deoxynucleotides. Therefore, it is necessary to retain this information before self-attention. The classic method of calculating position information is from learning (Takase and Okazaki 2019). There is no doubt that this method increases the additional time and memory overhead. Therefore, to improve the model’s efficiency, we use the formula to encode the position information and add the encoded data to the embedded vector. The encoding formula of location information is shown as Equation 9.


(9) $$PE_{\mathrm{pos}}=\{\begin{array}{cc} \;\text{sin}(\frac{\mathrm{pos}}{10000^{\frac{2i}{d_{\mathrm{model}}}}}) & i=2k\\ \;\text{cos}(\frac{\mathrm{pos}}{10000^{\frac{2i}{d_{\mathrm{model}}}}}) & i=2k+1 \end{array}$$

The formula originated from Vaswani *et al.*’s (2017) work. In the formula, *PE* represents the absolute position information after encoding, *pos* is the position, and 2*i* is the dimension of the embedded vector. Using this calculation method, not only the absolute position information $PE_{\mathrm{pos}}$ can be obtained, but also the relative position information $PE_{\mathrm{pos}+k}$ can be obtained through the linear transformation of $PE_{\mathrm{pos}}$Vaswani *et al.* (2017) proved that the effect of learned position information and calculated position information is not much different.

### 2.7 Multi-layer perceptron

We introduce a multi-layer perceptron to calculate the final Q value vector in the Q network (Q value can be regarded as a predicted SP score). The network consists of three linear layers. Parameters are shown in Supplementary Table S1. Since the output vector of the network is the normalized form of the Q value, it needs to be multiplied by the theoretical maximum Q value to convert it into the final Q value vector. The index of the Q value vector represents the actual action.

### 2.8 Model training

When the sequence state in the environment changes, environmental information is transmitted to the replay memory. When the number of quaternions stored in the replay memory reaches the minimum number of batches (128), $s_{t}$ and $a_{t}$ from the replay memory are transferred to the verification Q network, $s_{t+1}$ is transferred to the target Q network (the two Q networks have the same network structure), and then calculated $Q_{t}$ and $Q_{t+1}$ The two *Q* values respectively and corresponding $r_{t}$ from replay memory are transferred to the loss function to calculate loss, Update verification Q network parameters based on loss values When verifying Q network has been updated 128 times, synchronize parameter values to the target Q network.

To optimize the weight of the evaluation Q network, we use the mean square error as the loss function, and its expression is as follows:


(10) $$\mathrm{Loss}={\left(y-Q(s_{t},a_{t};\theta )\right)}^{2}$$

where $Q(s_{t},a_{t};\theta )$ is the action value $a_{t}$ under the state $s_{t}$. *y* indicates the max action value under the state $s_{t+1}$, called target Q value. The real trained network is the evaluated Q network in the training process. In contrast, the target Q network only copies the latest parameters from the evaluated Q network at regular intervals. In a sense, the structure composed of evaluating the Q network, target Q network, and loss function is considered an agent in this study because it can make decisions and adjust strategies.

## 3 Experimental results

We conducted multiple experiments on several datasets to evaluate DPAMSA. In particular, this experiment mainly answers the following questions:

- **RQ1**: Can our method get reliable results?
- **RQ2**: Can our method outperform the state-of-the-art MSA methods?
- **RQ3**: Are the positional encoding and self-attention mechanisms helpful to the final MSA?
- **RQ4**: How about the visualization results of the MSA?

### 3.1 Experimental setup

#### 3.1.1 Dataset

In the experiment, all sequences in datasets are a snippet from benchmarks such as OXBench (Raghava *et al.* 2003), BAliBASE (Thompson *et al.* 2005), SMART (Schultz *et al.* 2000), and PREFAB (Edgar 2004). These data can be download from GitHub. The datasets can be divided into five types, each type of dataset contains several sub-datasets, and each sub-dataset contains several sequences (3–6). The detail of all kinds of datasets is shown in Supplementary Table S2.

#### 3.1.2 Evaluation metrics

In this experiment, four metrics are used to evaluate the methods. These metrics are as follows: Sum-of-pairs (SP) score is the sum of all paired sequence scores, represented by Equation 1. CS is a standard criterion for evaluating alignment (Bawono *et al.* 2015). It is the quotient of exactly matched columns and the alignment length. The value range of the CS score is [0, 1] The higher the value, the better the alignment NASP: The number of optimal alignments based on SP score over one type of dataset NACS: The number of optimal alignments based on CS score over one kind of dataset.

#### 3.1.3 Baseline methods and hyperparameters settings

Seven commonly used MSA programs were selected in this control experiment. The algorithms used in these programs can be roughly divided into progressive alignment-based programs, iterative algorithm-based programs, and Hidden Markov model-based (HMM) programs. Specifically, the progressive alignment includes ClustalW (Thompson *et al.* 1994) and T-Coffee (Notredame *et al.* 2000). The iterative algorithm includes MAFFT (Katoh *et al.* 2002) and MUSCLE (Edgar 2004) the HMM-based methods includes ProbCons (Libin *et al.* 2019), Clustal Ω (Sievers *et al.* 2011), and MSAProbs (Liu *et al.* 2010).

Hyperparameters mainly include reinforcement learning parameters and relevant network parameters. The settings of these parameters in the experiment are shown in Supplementary Table S3.

### 3.2 Result of the method (RQ1)

In dataset experiments, the datasets, hyperparameters, and running environments used in the experiment are the same. After aligning the five types of datasets, the relevant scores in Table 1 are obtained. The first to fourth columns of the table lists the average SP score, average CS score, train time, and predict time.

**Table 1.** Relevant score of the results.

Datasets	Avg SP	Avg CS	Train time (h)	Predict time (ms)
1	150.00	0.65	0.75	2.55
2	280.96	0.56	1.16	2.80
3	390.08	0.46	1.4	3.15
4	601.92	0.39	1.70	3.25
5	324.89	0.62	1.85	3.45

The CS score is the ratio of the number of exactly matched columns to the length of the alignment, reflecting the overall quality of the alignment. Therefore, from the perspective of the average CS score, the first type and fifth types of datasets have better alignment quality. At the same time, the rate gradually decreases in the remaining three types. This downward trend has nothing to do with the method itself; the biggest reason is the sequence itself. Because the number of sequences increases, the nucleotide mismatch probability of each column gradually increases, which causes a decrease in the CS score. Because the number or length of sequences increases, the time of train and prediction gradually increases. The result data in Table 1 proves the method’s feasibility.

**Figure 3.**
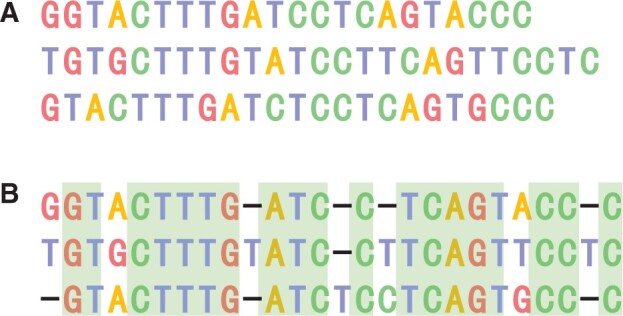
An alignment result calculated by DPAMSA. (**A**) The original sequence before being aligned. (**B**) Aan alignment result calculated by DPAMSA.

In addition, Fig. 3 shows an alignment of a sub-dataset in the first dataset type. In this figure, part (A) is the original sequence before being aligned, and part (B) is an alignment calculated by DPAMSA. It can be seen that DPAMSA can output an accurate alignment. Meanwhile, the alignment quality is also relatively good; among the 26 columns, 19 columns of deoxynucleotides are entirely matched. Finally, after calculation, the alignment’s SP and CS scores are 152 and 0.73.

**Figure 4.**
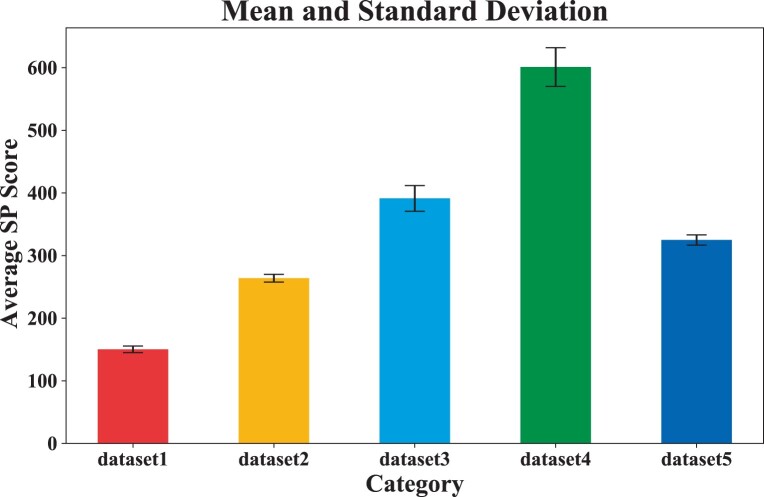
Mean and standard deviation of average sp scores in DPAMSA.

**Figure 5.**
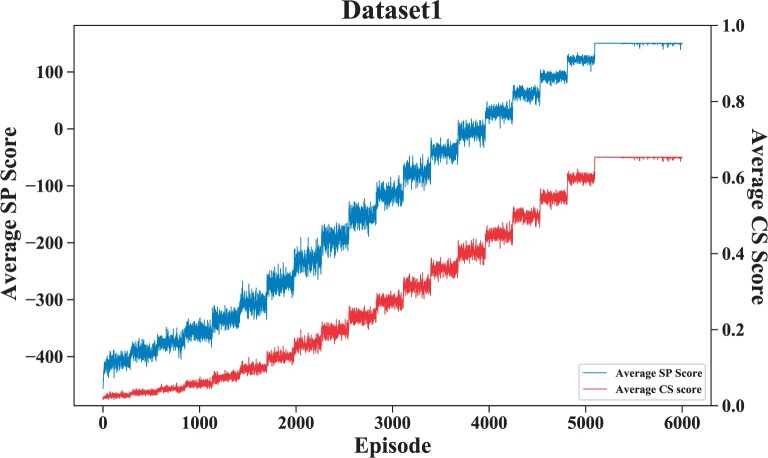
This figure shows the convergence of DPAMSA on dataset 1.

To verify the stability and convergence of the model, we repeated five dataset experiments, obtained the average SP score according to each aligned result, and calculated the mean and standard deviation of the five dataset experiments. Figure 4 shows the mean and standard deviation results. Figure 5 shows the variation of the average sp score during the training process.

As shown in Fig. 4, we can see that as the number of sequences in the dataset increases, the standard deviation increases, but within a controllable range. These results demonstrate that our model (OM) is stable.

As shown in Fig. 5, it can be seen that on dataset1, the episode is equal to around 5100, DPAMSA converges, and the average *SP* score is about 150. The oscillation is because the ϵ-greedy Greedy strategy randomly obtains action values with 1−ϵ probability.

### 3.3 Comparative experiment (RQ2)

Table 2 summarizes the comparison results between our method and all the baselines over the five type datasets regarding all four metrics NASP is the number of the optimal alignment over all sub-datasets in a specific type of dataset based on SP score, while the last NACS is based on CS score. The main observations from Table 2 are as follows:

**Table 2.** Comparative results of relevant score of the alignment.

MSA tools	Dataset1				Dataset2				Dataset3				Dataset4				Dataset5			
	Avg SP	Avg CS	NASP	NACS	Avg SP	Avg CS	NASP	NACS	Avg SP	Avg CS	NASP	NACS	Avg SP	Avg CS	NASP	NACS	Avg SP	Avg CS	NASP	NACS
Ours	150	0.65	49	48	280.96	0.56	43	41	390.08	0.46	35	35	601.92	0.39	13	16	324.89	0.62	8	8
ClustalW	108.56	0.57	0	0	189.28	0.45	0	0	245.44	0.36	0	1	449.92	0.33	0	0	319.56	0.61	0	0
T-Coffee	117.84	0.58	0	1	233.60	0.51	4	6	337.12	0.42	2	2	545.92	0.37	3	4	312.44	0.60	1	1
MAFFT	102.96	0.55	0	0	152.48	0.41	0	0	256.64	0.36	3	2	366.08	0.30	0	0	313.33	0.6	0	0
MUSCLE	98.72	0.54	0	0	196.00	0.45	0	0	280.80	0.38	0	0	444.32	0.33	0	0	318.67	0.61	0	0
ProbCons	128.64	0.61	0	0	254.72	0.53	1	2	375.36	0.44	7	5	588.80	0.39	8	5	310.56	0.6	0	0
ClustalΩ	56.96	0.46	0	0	92.00	0.34	0	0	120.80	0.27	0	0	203.52	0.22	0	0	304.44	0.59	0	0
MSAProbs	129.84	0.61	1	1	253.12	0.53	2	1	365.60	0.44	3	5	549.44	0.38	1	0	307.11	0.6	0	0

**Figure 6.**
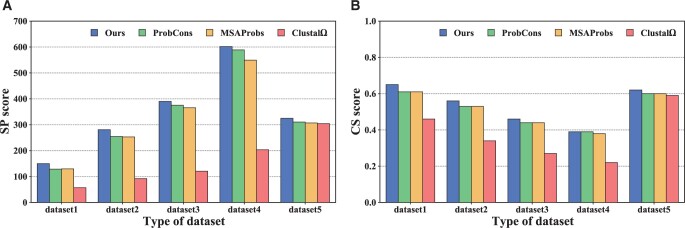
These figures show the comparisons of SP scores and CS scores among DPAMSA, ProbCons, Clustal Ω, and MSAProbs in (**A**) and (**B**)**,** respectively.

- Across all five types of datasets, DPAMSA can outperform all the competitors significantly. For example, the SP score of DPAMSA is 16%–163% higher than that of other programs over all datasets. Besides, DPAMSA is 7%–43% higher than others in the CS score. Moreover, even in the number of optimal alignments, our method also significantly improves.
- Most construct alignment based on guide trees for the progressive alignment-based programs (ClustalW and T-Coffee) and iterative alignment-based programs (MAFFT and MUSCLE). This indicates that the greedy nature of the construction process is easy to lead the methods to local optimum and lower accuracy DPAMSA adopts reinforcement learning, which can avoid the influence of greedy nature. For example, as shown in Table 2, DPAMSA is significantly ahead of MAFFT and MUSCLE in average SP and CS scores.
- For Hidden Markov model-based programs (Especially ProbCons and MSAProbs), use a posteriori probability to optimize the guide tree, significantly improving alignment accuracy. However, they adopt the idea of progressive alignment, and their accuracy still has room for improvement. For example, they are combined with Fig. 6 and Table 2, the average SP score of DPAMSA is 2%–17% higher than that of ProbCons and MSAProbs. At the same time, the average CS score is 0%–8% higher than these two programs The performance of Clustal Ω is so low because the application scenario of Clustal Ω is large-scale sequence alignment, which is not good at small-scale sequence alignment.
- Assuming that the significance level is .05 and taking the CS score as the sample, the significance of DPAMSA and the other seven programs are tested, respectively. The calculated *P* values are as follows: $2.95\times 10^{-9}$, $3.38\times 10^{-4}$, $1.10\times 10^{-13}$, $1.77\times 10^{-10}$, $6\times 10^{-2}$, $1.15\times 10^{-34}$, and $4\times 10^{-2}$ respectively. These *P* values show that our method significantly differs from other methods in the alignments except PronCons, while ProbCons is relatively tiny. More detailed derivations can be found in Table 2.

### 3.4 Model ablation (RQ3)

This section is an ablation experiment to determine our method’s utility of self-attention and positional encoding. We choose three models in this experiment, which are as follows: OM: This model keeps all modules described in Fig. 2B. Without Positional Encoding (WOPE): The model WOPE, i.e. the model WOPE, dropout layer, and normalization layer in Fig. 2B. Without All Modules (WOALL): The model without self-attention and positional encoding, i.e. the model only retains the embedding layer and multi-layer perceptron in Fig. 2B.

Table 3 summarizes the comparison results. Compared with the WOPE model, all other measures of WOALL are lower than those of WOPE except that the lowest CS score is slightly higher than WOPE. This result shows the feasibility of the self-attention mechanism in MSA. In addition, compared with OM, the six indicators of WOPE are much lower than ours. It is simply that the positional encoding can further improve the accuracy. Similarly, assuming a significance level of .05, the *P* values are .011 and .005, respectively. The *P* value further indicates a significant difference between the complete and the other two models. This experiment proves that applying self-attention and positional encoding in MSA is feasible. Each of them can improve the accuracy of alignment to a certain extent.

**Table 3.** Relevant score of OM, WOALL, and WOPE.

Model	Min SP	Max SP	Avg SP	Min CS	Max CS	Avg CS
OM	336	1124	601.92	0.19	0.70	0.39
WOPE	−184	1020	362.08	0.07	0.63	0.30
WOALL	−288	948	332.48	0.08	0.59	0.29

### 3.5 Case and visualization (RQ4)

**Figure 7.**
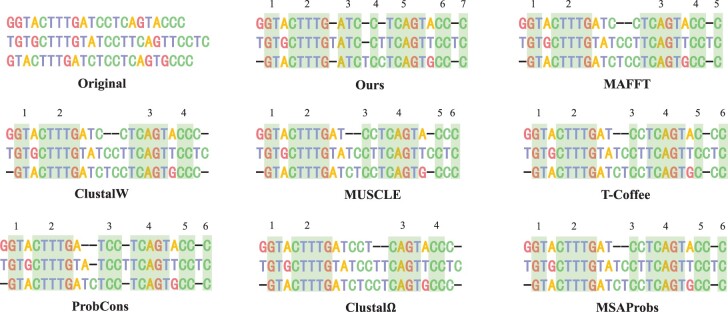
Visualization results of DPAMSA and seven state-of-the-art methods. The number of homologous regions are marked on the top of each alignment. It can be seen that DPAMSA exceeds other programs in the number of homologous regions.

In addition to these metrics mentioned above, we also found that our method can detect more homologous regions from the alignment. For example, Fig. 7 shows the alignments in various ways on a sub-dataset. In this figure, the second alignment was computed by DPAMSA. It can be seen from it that DPAMSA found 7 homologous regions, while other programs only found 4–6 of them. In addition, the way also detects homologous regions that other programs cannot see, such as region [*ATC*, *ATC*, *ATC*] in the second alignment.

## 4 Conclusion

This paper presents a DRL-based method with natural language process techniques to solve the MSA problem. We argue that the sequence feature is critical to enhancing the accuracy of the alignment. We introduced a self-attention mechanism to extract the feature of the current alignment and then utilized positional encoding to improve the reliability of the extracted feature. Finally, the feature was input into a multi-layer perceptron to predict the weight of the action in reinforcement learning. To our knowledge, DPAMSA is the first technique that exploits self-attention and positional encoding on the sequence alignment. We conducted extensive experiments on five datasets and demonstrated that this method could remarkably outperform several state-of-the-art competitors in MSA. Besides we also carried out the ablation experiment, which proves the effectiveness of the self-attention mechanism and positional encoding. In the future, we plan to optimize the architecture of the current model to reduce the complexity. We believe this paper would provide a novel and clear insight to the researchers engaging in MSA.

## Supplementary Material

btad636_Supplementary_Data

## Data Availability

The data underlying this article are available at https://github.com/ZhangLab312/DPAMSA.
