# Supplementary material for: Multiple sequence alignment based on deep reinforcement learning with self-attention and positional encoding
Source: Bioinformatics. 2023 Oct 19;39(11):btad636. doi: 10.1093/bioinformatics/btad636 (PMC10628385; doi:10.1093/bioinformatics/btad636)
Supplement: btad636_Supplementary_Data [file btad636_supplementary_data.docx]

**Supplementary Information**

Supplementary Table 1. The parameters of multi-layer perceptron.

| Name | Input Size | Ouput Size | Activate Function |
| --- | --- | --- | --- |
| Linear1 | *d_model_* X *n* X *L* | 1028 | LeakyReLU |
| Linear2 | 1028 | 512 | LeakyReLU |
| Linear3 | 512 | 2*^n^*-1 | Tanh |

Supplementary Table 2. Detail of all datasets.

| Type | Sub-datasets | Sequences in sub-datasets | Average sequence lenth | Total sequences |
| --- | --- | --- | --- | --- |
| 1 | 50 | 3 | 30 | 150 |
| 2 | 50 | 4 | 30 | 200 |
| 3 | 50 | 5 | 30 | 250 |
| 4 | 25 | 6 | 30 | 150 |
| 5 | 9 | 3 | 60 | 27 |

Supplementary Table 3. Hyperparameters setting.

| Hyperparameter | Value |
| --- | --- |
| *γ*, discount factor. | 1.0 |
| *ε_i_*, initial *ε* in *ε*-greedy strategy. | 0.8 |
| *ε_f_* , final *ε* in ε-greedy strategy. | 0.0 |
| *τ_ε_*, decrement iteration of *ε*. | 150(episodes) |
| *∆_ε_*, reduction value of *ε*. | 0.05 |
| d_model_, dimension of the embedded vector. | 64 |
| d_k_, dimension of the vector key and query. | 64 |
| d_v_, dimension of the vector value. | 64 |
| α, learning rate in Adam optimizer. | 0.0001 |
| episode, number of iterations of training. | 3000~10000 |
| replay memory size. | 1000 |
| update iteration of target Q network. | 128(time-steps) |
| batch size. | 128 |


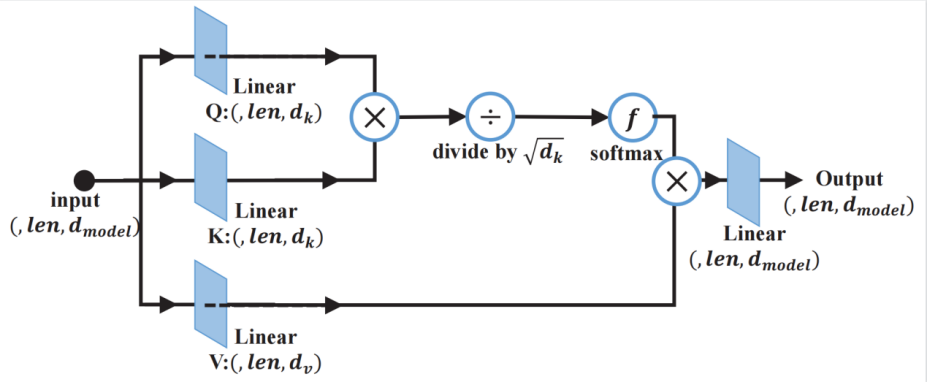


Supplementary Figure 1. This figure illustrates the structure of self-attention.
